# Research on the Mechanism of Hydrogen Plasma Heating and Reduction of Acidic Pellets

**DOI:** 10.3390/ma19061269

**Published:** 2026-03-23

**Authors:** Zihao Fan, Xiaoping Zhang, Chuanwen Geng, Xingyue Jin, Lin Li, Peng Zhao, Baoliang Wen, Jialong Yang

**Affiliations:** 1 School of Metallurgical Engineering, Anhui University of Technology, Ma’anshan 243032, China; zihaofan@ahut.edu.cn (Z.F.); wenbaoliang@ahut.edu.cn (B.W.); jialongyang@126.com (J.Y.); 2 Technology Center, Ma’anshan Iron and Steel Co., Ltd., Ma’anshan 243003, China; 3 School of Carbon Neutrality Science and Engineering, Anhui University of Science and Technology, Huainan 232001, China; 4 Institute of Plasma Physics, Chinese Academy of Sciences, Hefei 230088, China; xingyue.jin@ipp.ac.cn (X.J.); linli@iim.ac.cn (L.L.); pzhao@ipp.ac.cn (P.Z.)

**Keywords:** hydrogen plasma, non-transferred arc, smelting reduction, high-purity iron, reduction kinetics, reduction rate

## Abstract

Hydrogen plasma heating, a unique method for heating and reducing iron ore, is distinguished by its high heat, rapid reduction, and high efficiency, making it a promising technique in the metallurgy field. In this study, a non-transferred arc plasma heating system was used with Ar-H_2_ as the working gas and acidic pellets as the raw material. The microstructures and elemental distributions of the slag and iron phases during the reduction process were examined using electron microscopy and energy-dispersive X-ray. The variation patterns of Fe-containing phases in the reduction products were found using X-ray diffraction and full-spectrum fitting refinement. The conversion rate of the oxidized pellets and the deoxidation conversion rate per area were estimated for various gas flow rates and reduction times. A reaction kinetics model was also used to study the reaction controlling step. The results showed that during the reduction process, with an H_2_ flow rate of 4.5 L min^−1^ and a 40 min reduction, the conversion(α) reached 99.89% and the purity of the reduced metallic iron reached 99.9%, achieving the industrial-grade 3N standard. Si and Al in the melt bath generated fayalite (Fe_2_SiO_4_) and hercynite (FeAl_2_O_4_) with Fe_x_O. The deoxidation conversion rate per unit area was 1.11 g (cm^2^ min)^−1^. A three-dimensional diffusion-controlled model was used to describe the reduction process, and the mechanism function was 2/3(1 + α)^3/2^[(1 + α)^1/3^]^−1^. The values of the reduction reaction rate constant (K) were 12.6 × 10^−2^ s^−1^ and 12.8 × 10^−2^ s^−1^ when the flow rates of H_2_ gas were 3 and 4.5 L min^−1^, respectively. The apparent activation energy was 21.9 kJ mol^−1^. The empirical equation for the specific reduction rate was calculated as ln r = −2637.5/T − 0.407.

## 1. Introduction

Currently, greenhouse gas emissions pose a significant threat and challenge to sustainable human development. Reducing carbon emissions and promoting the application and development of new energy sources have become a global consensus [1,2]. To respond to the trend of the “dual carbon” strategy (carbon neutrality and carbon peak), developing green, innovative, and low-carbon ironmaking technologies has become an important research direction in the development of the metallurgical industry.

Carbon and hydrogen can undergo gas-based and molten reduction in iron ore reduction, but hydrogen can greatly reduce greenhouse gas CO_2_ emissions. Thus, the global steel industry is actively exploring various low-carbon iron production technologies, and replacing carbon with hydrogen in iron ore reduction is regarded as an effective and sustainable method [3]. Significant investments have been made in research projects such as Sweden’s HYBRIT project (launched in 2016) and the United States’ ROSIE program (launched in 2024) [4]. SuSteel, a hydrogen smelting project developed by Voestalpine, is considered to be a highly innovative and developmental new smelting technology that uses hydrogen plasma in metallurgical production processes. HPSR furnaces have been engineered and adapted to improve the innovative characterization in K1-MET GmbH’s pilot HPSR plant at Donawitz, Austria [3]. Laboratory-scale experiments conducted at the Pyrometallurgy Laboratory, Indonesia, produced metallic iron from goethite in 120 s, ferronickel in 90 s, and ferrochrome (around 50% Cr) in 240 s [5]. Hydrogen plasma presents a feasible substitute for carbon-neutral iron production, potentially enhancing the reduction rate of Fe_x_O significantly [6,7,8,9]. In theory, H_2_O formation inhibits the formation of carbon-containing gases.

Hydrogen plasma smelting reduction (HPSR) is an innovative ironmaking technique that melts and reduces iron ore at the same time. This technology was first proposed by the University of Leoben in 1992, and Hiebler and Plaul [10] determined that it might be able to achieve more economical and ecologically friendly green steel manufacturing, with economic costs reduced by 20% compared to those of previous methods. Stokes [11] found that a 100% metal conversion of Fe_3_O_4_ could be achieved under a mixed gas flow of H_2_ and He. Behera et al. [12] employed a non-transferred arc plasma torch with continuous feeding, and they demonstrated a conversion rate of 95.5% in a 7 kg large-scale experiment for the first time. Nakamura et al. [13] discovered that the quantity of fragmented hydrogen atoms in the overheated gas created by the hydrogen plasma resulted in a hydrogen usage rate of 50–70%, which exceeded the maximum value for molecular hydrogen. Naseri et al. [14] conducted experiments on the smelting reduction of magnetite powder using thermal hydrogen plasma and obtained high-purity iron with an iron content of 99.7%. The total conversion rate was around 80%, with a maximum deoxygenation rate of 0.7 g min^−1^.

The HPSR reactions described above were achieved using a transferred arc direct current (DC) plasma furnace. There are DC plasma torches that utilize CO_2_ and CH_4_ as carrier gases. These technologies provide the highest energy density, a sufficient amount of H for the reduction process, and not only avoid formation but also use greenhouse gases as a feedstock. In this type of furnace, the cathode is a graphite or tungsten electrode, and the anode is a generally copper or steel crucible, which should be placed on a graphite plate. This allows samples to be reduced at high temperatures, which influences the final conversion rate computation. Meanwhile, the reactants must be conductive substances or be conductive in the molten state, thus serving as one of the electrodes of the plasma [15]. Non-transferred arc plasma torches, however, can avoid this limitation because the materials to be heated do not have to be conductive, resulting in safer operation [16,17]. This sort of torch generates a plasma between the electrodes inside the torch body, which holds a considerable amount of energy and reaches temperatures of up to 10^4^ K [18,19]. When processing medium-grade ores, mixing Ar and H_2_ in a certain ratio and ejecting them from the torch makes the plasma procedures more cost-effective than hydrogen direct reduction–electric arc furnace (HDR-EAF) steelmaking [20]. Behera et al. [12] and other researchers proposed the ratio of the crucible bath height to its diameter as a significant parameter influencing the plasma fluid flow and final conversion rate. When the hydrogen flow rate is between 10 and 20% and the bath height-to-diameter ratio is between 0.2 and 0.25, excited-state hydrogen particles can be effectively mixed with molten iron oxides, enhancing the conversion rate and increasing the hydrogen utilization [21,22,23].

The study of non-transferred arc HPSR of iron ore is still in its early stages. The reduction process and phase product modifications have not been completely studied, particularly the reaction mechanism and reduction kinetics. This study, based on the high calorific value, rapid heating, and strong separation effect of hydrogen plasma, aimed to reduce the contents of the impurity elements Si and Al, improve the purity of metallic iron, explore the phase transformation characteristics of non-transferred arc hydrogen plasma-reduced iron ore, and achieve efficient and rapid green reduction. By using acidic pellets from steel plants as experimental materials, the influences of the reduction time and gas flow on the reduction process were examined in this study to further elucidate the reaction kinetics and mechanism through which non-transferred arc DC hydrogen plasma reduces pellet ore. The results provide a theoretical basis for green and low-carbon smelting processes.

## 2. Materials and Methods

### 2.1. Experimental Materials

**Figure 1:**
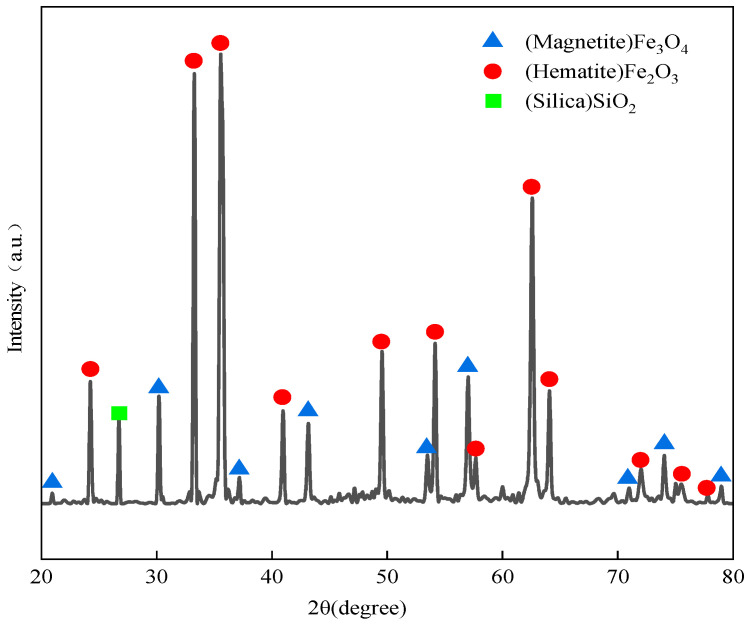
X-ray diffraction (XRD) patterns of acidic pellets.

The raw materials used in the experiment were acidic pellets provided by Maanshan Steel Research and Technology Center (Ma’anshan, China), with an average particle size of 12 to 16 mm. Elemental content analysis and oxide content analysis of the acidic pellets were conducted using XRF. The chemical composition of the acidic pellets is shown in Table 1. The total iron content reached 69.35%, with the main impurity elements Si and Al, while the content of other trace elements was below 0.2%. The main oxide component was iron oxide Fe_2_O_3_, and the calculated binary basicity R(w(CaO)/w(SiO_2_)) was 0.09, where R is binary basicity and w is mass fraction. The pellets were cut along their diameters into hemispheres with smooth surfaces, and XRD was used to obtain diffraction spectra of the iron phases and SiO_2_, as shown in Figure 1.

### 2.2. Experimental Apparatus and Research Methods

**Figure 2:**
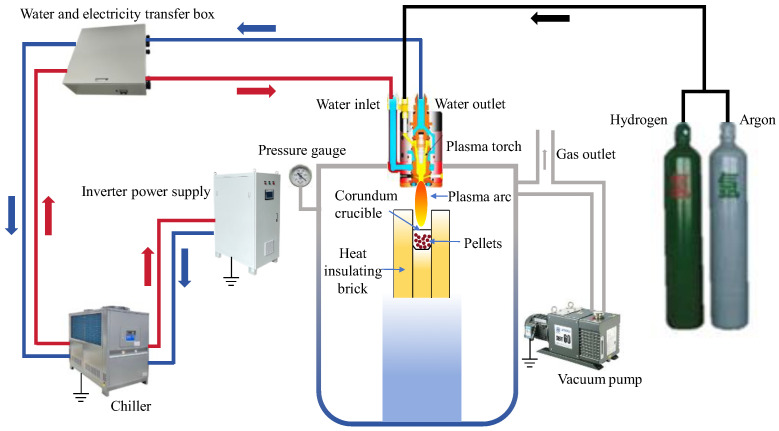
Experimental apparatus.

The experimental apparatus mainly consisted of the following components: a plasma torch, vacuum chamber, water cooling circulation system, gas delivery system, control system, inverter power supply system, and vacuum pump system. The plasma torch used was the SG-100 model manufactured by Praxair, Danbury, CT, USA. The water cooling circulation system, control system, and inverter power supply system were manufactured by Shanghai Xiuma Spraying Machinery Co., Ltd. (Shanghai, China). The vacuum pump system was the BSV60 model manufactured by Busch Vacuum (Ningbo, China). The experimental apparatus is shown in Figure 2. The experimental gases were high-purity argon (99.9%) and high-purity hydrogen (99.9%) from Hengxing Gas Co., Ltd. (Hefei, China).

First, 50 g of acidic pellets was weighed with a balance and then placed on a corundum crucible in a vacuum chamber. The vacuum chamber was then sealed and evacuated to −0.07 MPa, after which Ar was introduced until the pressure inside the chamber reached 0.01 MPa. The current of the plasma torch was set to 300 A, with the plasma torch nozzle 90 mm from the surface of the acidic pellet sample. To prevent the iron ore from remaining in an unmelted state during the reduction process, the plasma was ignited in an Ar atmosphere for 2 min to fully melt the acidic pellets to the liquid state, then hydrogen was added to the plasma smelting reduction atmosphere until its concentration reached 15%. Table 2 shows the experimental parameters for hydrogen plasma reduction. After the reduction was complete, Ar was introduced to stratify the slag and iron in the crucible, and the plasma torch was cooled.

After reduction, the sample was reweighed using a balance to determine the amount of oxygen removed from the iron oxides by H_2_ reduction. The mass of recovered metal and slag was calibrated through multiple weighings. The reduced sample was then cut and crushed along the cross-section using a cutting machine to separate the iron and slag. The slag was polished to a smooth surface, and X-ray diffraction (XRD) was used to identify the phase composition of the slag (XRD system model: Rigaku SmartLab SE Cu target from Rigaku Corporation, Tokyo, Japan). The changes in the Fe phase contents in the slag were determined by full-spectrum refinement using the software Jade 9. Scanning electron microscopy–energy-dispersive X-ray spectroscopy (SEM-EDS; SEM system model: ZEISS GeminiSEM 360 from Carl Zeiss Microscopy GmbH, Jena, Germany) was used to evaluate the microstructure and composition of the slag–iron interface. In the experiment, the average temperature of the flame flow region ejected from the center of the plasma torch ranges from 4300 °F to 10,300 °F [24], which was higher than the breakdown temperature of Fe_2_O_3_ (1113 K) in air. However, the partial pressure of oxygen in the argon environment was lower, which reduced the decomposition temperature [17]. Some of the Fe_2_O_3_ on the surfaces of the pellets evaporated, the loss of dust/debris or the portion carried out of the crucible by gas flow, and the mass loss was determined through experiments with a blank group, represented by M_0_ in Equation (1). The blank group experiments were identical to those of the experimental group except for the absence of the reducing agent hydrogen. The extent of the iron reduction was calculated as follows [18]:

(1) $$\alpha =\frac{M_{1}-M_{2}-M_{0}}{O_{ore}},$$

where α is the conversion of the sample (%), M_1_ is the initial total mass of the unreduced sample (g), M_2_ is the total mass of the reduced sample after the reaction (g), M_0_ is mass loss of a crucible with acidic pellets that underwent the smelting process with argon gas (g), and O_ore_ is the content of oxygen bound with Fe in the ore (g).

## 3. Experimental Results and Discussion

### 3.1. Macroscopic Analysis of Acidic Pellets Reduced by Hydrogen Plasma

**Figure 3:**
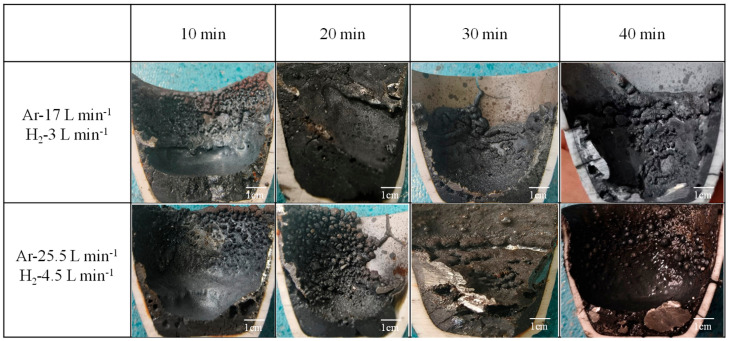
Macroscopic photographs of test samples with different gas flow rates and reduction times.

After the reaction sample had naturally cooled, the crucible was cut in half vertically with a cutting machine. Figure 3 depicts macroscopic images of the test samples obtained at various gas flow rates and reduction times. The figure shows the melt bath after reduction with hydrogen plasma, which had a concave-downward arc shape. During the heating and melting stage, high-energy excited-state hydrogen particles first reduced the iron oxides on the surface of the melt bath to produce metal. After 10 min of reaction at an H_2_ flow rate of 3 L min^−1^, a thin metallic layer formed on the surface of the melt bath, with part of the reduced metal sticking to the crucible’s inner wall. The majority of the reddish-brown Fe_2_O_3_ on the pelletized ore’s surface was removed. Extending the reaction time to 30 min improved the conversion rate, with a tiny amount of metallic luster material evenly distributed on the surface of the melt solution and some micropores in the spaces that separated the slag. However, the metal’s thickness was minimal. At 40 min, large blocky metal enrichments formed on the bottom and side walls of the crucible, with a smooth surface. When the hydrogen flow rate was increased to 4.5 L min^−1^ for 20 min, the impact of the gas flow was severe, generating dense spherical substances on the inner wall of the crucible and resulting in poor separation between the slag and metal. When the reaction time was increased to 40 min, the separation between the metal and slag was visible, with a considerable amount of metal accumulating at the bottom. Furthermore, the metal surface was smooth, with only a small amount of oxide slag present within the crucible, corresponding to a relatively reducing effect.

### 3.2. Phase Composition Analysis of Acidic Pellets Reduced by Hydrogen Plasma

**Figure 4:**
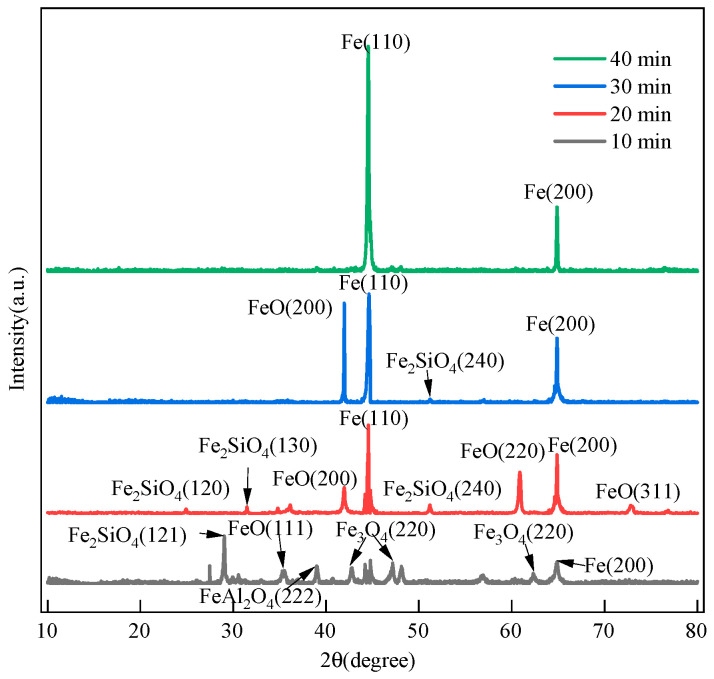
XRD patterns of melted phase with hydrogen flow rate of 4.5 L min^−1^.

For the samples that reacted under different conditions, we selected areas with a clear stratification of the slag and iron phases for cutting. Macroscopically, we observed different color characteristics of the iron oxides in these areas. Figure 4 shows the compositions of the slag–iron phases, as measured by X-ray diffraction, for the samples with a reduction gas flow rate of 4.5 L min^−1^. The figure shows that after 10 min of reaction, a small amount of Fe_2_O_3_ had evaporated, and the remaining Fe_2_O_3_ had gradually converted into Fe_3_O_4_, FeO, and a three-phase Fe system. The elements Si and Al reacted with Fe_x_O to form FeAl_2_O_4_ and Fe_2_SiO_4_, respectively. With the high-speed jet of hydrogen plasma, excited-state hydrogen atoms in the melt bath, termed [H], were produced in large quantities. After 20 min of reaction, a significant amount of [H] had reached the surface and interior of the melt bath. A huge amount of [H] contacted the active sites of Fe_x_O, accelerating the reaction and creating a significant amount of reduced iron. The inadequate fluidity of Fe_2_SiO_4_ hindered its reduction upon contact with [H]. After 30 min of reaction, the majority of FeAl_2_O_4_ and Fe_2_SiO_4_ in the melt bath had been reduced by [H], the majority of the FeO reduction had been completed, and a minimal quantity of Fe_2_SiO_4_ persisted in the melt bath. After 40 min of reaction, the diffraction spectrum only contained peaks corresponding to Fe. At this time, the reduction to metallic iron was complete. However, due to the vigorous swirling of the plasma gas flow, parts of the Fe_2_SiO_4_, FeAl_2_O_4_ and Fe phases were physically blown out by the high-speed jet impact.

### 3.3. Microscopic Morphology and Composition Analyses of Acidic Pellets Reduced by Hydrogen Plasma

**Figure 5:**
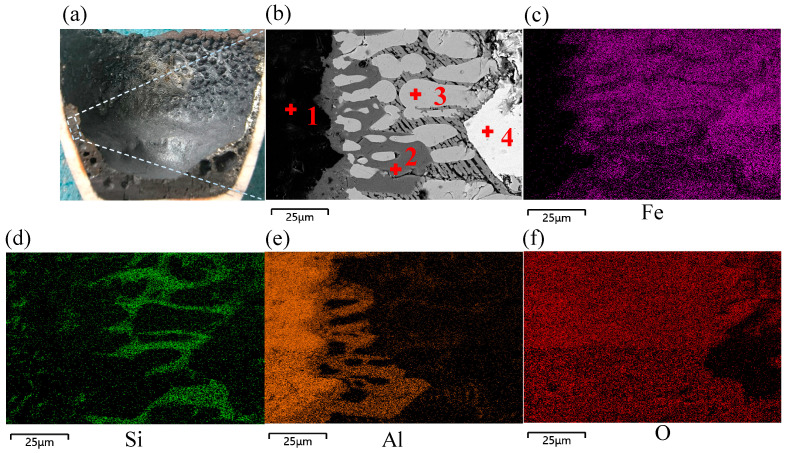
Microscopic morphology of cross-section of reduced sample with hydrogen flow rate of 4.5 L min^−1^ at 10 min: (**a**) reduced sample cross-section, (**b**) microscopic morphology, (**c**) distribution of Fe, (**d**) distribution of Si, (**e**) distribution of Al, (**f**) distribution of O.

SEM-EDS was used to study the microstructures and elemental distributions of the samples reduced with a plasma gas flow rate of 4.5 L min^−1^. The macroscopically selected sampling location exhibited a strong slag iron stratification effect, and microscopically, transitional reduction products were identified at the boundaries of slag iron phases, showing that the metallic iron reduction was a progressive process. In the first step, there were two intermediate products, FeO and Fe_3_O_4_, and as the reduction process progressed, a huge amount of metallic iron accumulated at the bottom of the crucible. The microstructural morphologies of elemental Fe spots were analyzed using SEM after various reduction durations to detect changes in the contents of elemental Fe and impurity elements. Figure 5 depicts the reduced sample’s cross-section and microstructure with a hydrogen flow rate of 4.5 L min^−1^ at 10 min.

**Figure 6:**
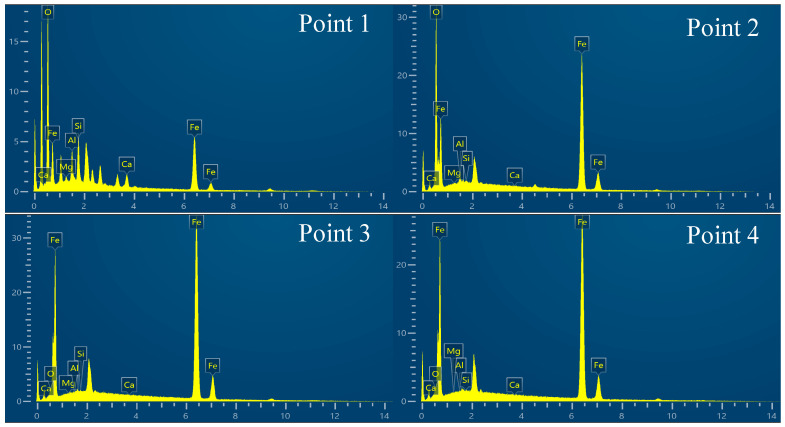
Energy spectra of points 1 to 4 in Figure 5.

The dashed box area in Figure 5a was observed using SEM to analyze its microstructure, and mapping analysis was conducted on the elemental contents of Fe, O, Si, and Al. Metallic iron accumulated at the surface of the melt bath, and the impurity elements Al and Si were completely separated from the generated elemental Fe. The majority of the Al was accumulated at the wall of the crucible, with Si forming a minor amount of dark striated Fe_2_SiO_4_ in the spaces between FeO and Fe_3_O_4_. Figure 6 shows the energy spectrum of points 1 to 4 in Figure 5b, and Table 3 shows the corresponding elemental analysis results.

**Figure 7:**
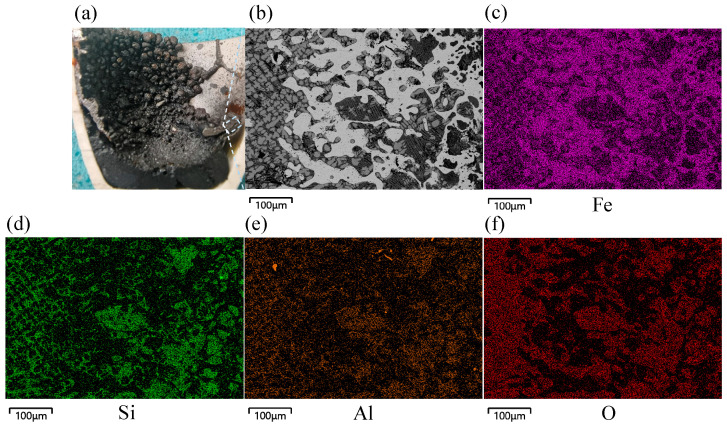
Microscopic morphology of cross-section of reduced sample with a hydrogen flow rate of 4.5 L min^−1^ at 20 min: (**a**) reduced sample cross-section, (**b**) microscopic morphology, (**c**) distribution of Fe, (**d**) distribution of Si, (**e**) distribution of Al, (**f**) distribution of O.

Figure 7 shows the reduced sample’s cross-section and microstructure with a hydrogen flow rate of 4.5 L min^−1^ at 20 min. The produced elemental Fe was dendritically enriched with a significantly increased content. The impurity elements Si and Al generated striated FeAl_2_O_4_ (black) and Fe_2_SiO_4_ (dark gray) that were interspersed with the elemental Fe. This effect was due to the high-speed impact produced by the high-temperature plasma jet, which promoted adequate stirring of the melt solution while preventing total separation of the slag and iron. This prevented the enriched elemental Fe from depositing quickly at the bottom of the crucible, and it came into contact with the rising FeAl_2_O_4_ and Fe_2_SiO_4_, thus forming a mixed state. On the left side, the gray unreduced Fe_x_O was linearly and orderly arranged, and it was in close contact with the elemental Fe. These results indicated that the reduced-state [H] content at this location was low, and [H] had not diffused to the reaction interface in time to participate in the reaction.

**Figure 8:**
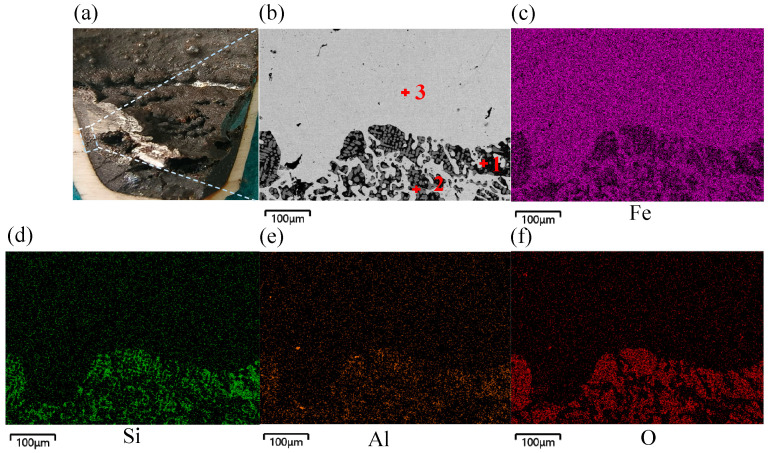
Microscopic morphology of cross-section of reduced sample with hydrogen flow rate of 4.5 L min^−1^ at 30 min, (**a**) reduced sample cross-section, (**b**) microscopic morphology, (**c**) distribution of Fe, (**d**) distribution of Si, (**e**) distribution of Al, (**f**) distribution of O.

**Figure 9:**
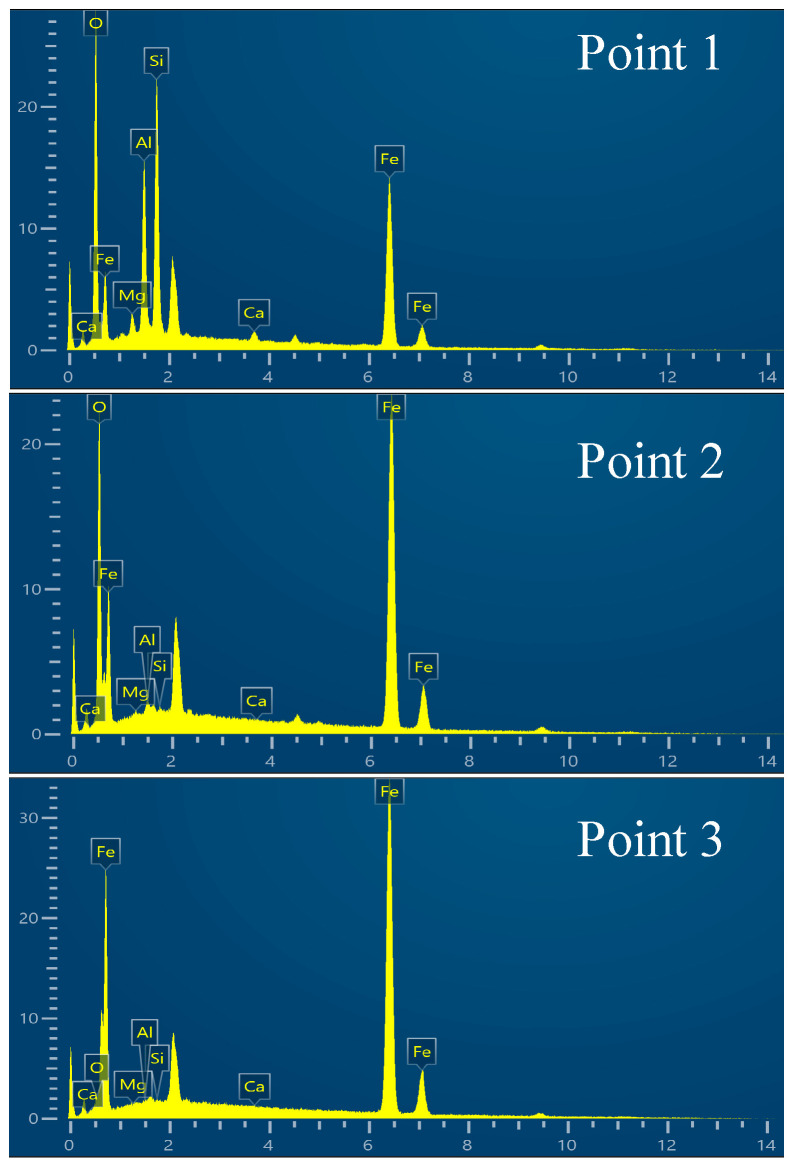
Energy spectrum of points 1 to 3 in Figure 8.

Figure 8 shows the cross-section and microstructure of the reduced sample with a hydrogen flow rate of 4.5 L min^−1^ at 30 min. The micrograph clearly shows that elemental Fe and the slag phase were well layered. The interface was wavy due to oscillations in the melt pool. The elemental Fe that was created was highly concentrated near the bottom of the crucible. Additionally, XRD phase analysis revealed that the main impurity in the slag phase was Fe_2_SiO_4_. At the same time, the predominant unreduced iron oxide present was Fe_x_O, which was found in the elemental Fe phase as granular particles. Figure 9 shows the energy spectrum of points 1 to 3 in Figure 8b, and Table 4 shows the corresponding elemental analysis results.

**Figure 10:**
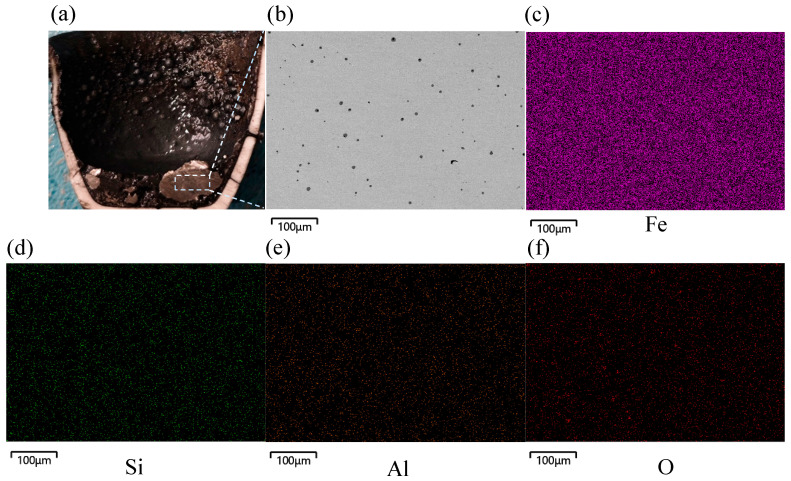
Microscopic morphology of cross-section of reduced sample at 40 min: (**a**) reduced sample cross-section and (**b**) microscopic morphology, (**c**) distribution of Fe, (**d**) distribution of Si, (**e**) distribution of Al, (**f**) distribution of O.

Figure 10 illustrates the cross-section and microstructure of the reduced sample with a hydrogen flow rate of 4.5 L min^−1^ at 40 min. Macroscopically, the metallic iron was evenly distributed at the bottom of the crucible, as shown by the square in Figure 10a. EDS scanning showed that most of the impurity elements, such as Si and Al, were eliminated. Only a small amount of Si was present as black granular particles in the Fe.

### 3.4. Analysis of Conversion and Conversion Rate of Acidic Pellets by Hydrogen Plasma

**Figure 11:**
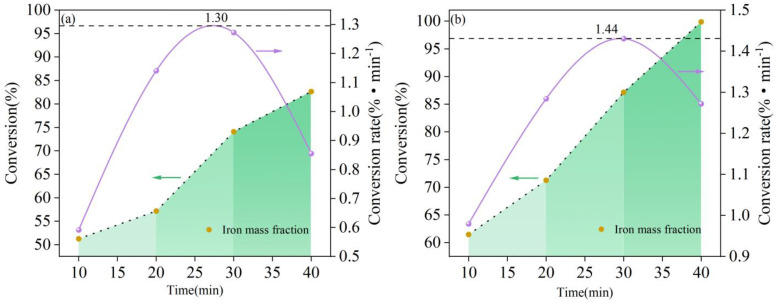
Effect of gas flow rate and time on conversion rate and reduction rate: (**a**) Ar flow rate of 17 L min^−1^, H_2_ flow rate of 3 L min^−1^; (**b**) Ar flow rate of 25.5 L min^−1^, H_2_ flow rate of 4.5 L min^−1^.

The phase composition was determined using Jade (6.0) based on the full-spectrum fitting refinement analysis of the reduction products’ diffraction spectra, and mass conservation before and after acidic pellet reduction was used to calculate the conversion rate for different reduction gas flow conditions and times. Figure 11 shows the conversion rate, which was derived by taking the derivative of the conversion over time. The first conversion rate in the figure is calculated based on the conversion at T = 0 s, while the others are calculated with respect to the conversion of the previous moment. The dashed and solid lines show the trends in the conversion and conversion rate, respectively. The figure clearly shows that the transformation process of iron was divided into three stages. In the first stage (T < 20 min), the reaction proceeded slowly, and the conversion had a mild upward tendency. During this stage, hydrogen only participated in the reduction reaction on the surface of the melt bath and did not enter deeply into its interior. As the concentration of hydrogen increased in the second stage (20 min < T < 30 min), the gas flow’s stirring effect on the melt bath strengthened, allowing hydrogen to enter the interior and expand the reduction area. This accelerated the conversion rate and resulted in a clear upward trend in the conversion. The iron generated during the reduction had a higher mass density than the remaining Fe_x_O and sank to the bottom of the crucible. In contrast, the remaining Fe_x_O was dispersed in the top slag. This distribution of metallic iron and slag was the same as that found in a previous study [25]. The conversion rate dropped sharply in the third stage (T > 30 min). At this time, the majority of the iron oxides had been reduced, the reactant concentration had decreased, the interaction between hydrogen particles and iron oxides had deteriorated, and the conversion rate had decreased.

Table 5 displays the maximum conversion rate and rate for various reducing atmospheres and reduction times, as well as the deoxygenation rate per unit surface area during the reduction process. The data indicates that at a gas flow rate of 3 L min^−1^, the maximum reduction rate was merely 1.30% min^−1^. Increasing the gas flow rate to 4.5 L min^−1^ raised the concentration in the reducing environment, resulting in adequate kinetic conditions for the reaction. The reactivity of excited-state hydrogen particles increased, resulting in a maximum reduction rate of 1.44% min^−1^ and a conversion rate of 86.6%. This was significantly higher than the value of 69.72% at the gas flow rate of 3 L min^−1^ and higher than the value of 82.5% reported by Naseri et al. when Fe_2_O_3_ powder was continuously fed into a DC plasma arc furnace [26]. The mass loss during the reduction process corresponds to the mass loss of oxygen elements combined with iron. Therefore, the mass loss of oxygen elements per area of melt bath is used to define the deoxidation rate per area. When comparing the experimental deoxygenation reduction rate per unit area to those in other experiments, assuming that the spherical cavity at the time of the plasma jet impacting the melt served as the reaction interface, the deoxygenation rates per unit surface area of the Ar-H_2_ plasma at H_2_ flow rates of 3 and 4.5 L min^−1^ reached 0.87 and 1.11 g (cm^2^ min)^−1^, respectively. These results were higher than the 0.53 g (cm^2^ min)^−1^ deoxygenation rate per unit area found by Kamiya et al. [27]. This difference is significant, indicating that the high temperature and high energy of the H_2_ plasma improved the thermodynamic and kinetic conditions of the reduction reaction.

### 3.5. Analysis of Reaction Kinetics Model for Hydrogen Plasma Reduction of Acidic Pellets

To thoroughly investigate the limiting steps in the reduction of acidic pellets using hydrogen plasma, we performed integral fitting of the mechanism function to analyze the kinetics involved in the reduction process of iron oxides by hydrogen plasma. Firstly, take the time derivative of α to establish the relationship between the rate of transformation and time and temperature, as shown in Equation (2), and then Equation (2) is transformed and integrated as shown in Equation (3). The rate constant is then obtained by integrating dα/f(α), using the values of α, and performing a linear regression of the integrated function values with time. The equations are shown as follows:

(2) $$\frac{d\alpha }{dt}=k\left(T\right)f\left(\alpha \right)$$

(3) $$G\left(\alpha \right)=\int_{0}^{\alpha }\frac{d\alpha }{f\left(\alpha \right)}=\int_{0}^{t}k\left(T\right)dt=k\left(T\right)t,$$

where f(α) is the differential form of the reaction mechanism function (%), G(α) is the integral form of the reaction mechanism function, α is the conversion rate (%), t is the reaction time (s), and k(T) is the reaction rate constant (s^−1^).

**Figure 12:**
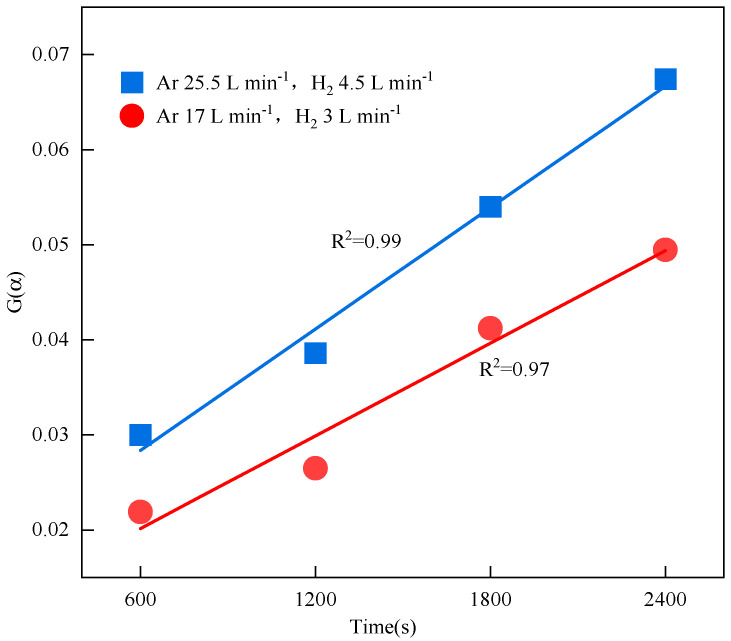
Three-dimensional diffusion model fitting results.

By comparing the common chemical reaction kinetics models used by previous researchers [22], including the diffusion-controlled model, power law model, shrinking core model, chemical-reaction-controlled model, and stochastic nucleation and growth model, it was found that under the experimental conditions of this study, the three-dimensional diffusion-controlled model showed a good linear fitting effect with the reduction time, achieving a coefficient of determination (R^2^) value of 0.99 at an H_2_ flow rate of 4.5 L min^−1^. Figure 12 shows the fitting results for the appropriate kinetic mechanism function, 2/3(1 + α)^3/2^[(1 + α)^1/3^ − 1]^−1^. Figure 12 also clearly shows that, as the gas flow rate increased, the reaction rate constant increased dramatically. When the gas flow rate was 3 L min^−1^, the reaction rate constant was 12.6 × 10^−2^ s^−1^. However, increasing the gas flow rate to 4.5 L min^−1^ decreased the reaction rate constant to 12.8 × 10^−2^ s^−1^. This reaction rate constant was several orders of magnitude greater than the apparent rate constant of 1.6 × 10^−6^ s^−1^ found by Ban-Ya et al. [28] using a SiC resistance furnace at 1673 K for the melting reduction of iron oxides. This disparity primarily stemmed from two factors. On the one hand, in this experiment, the plasma flame flow was arc-shaped, only mixing and sweeping the surface of the molten oxide inside the crucible and not diffusing deeply into the melt bath, which to some extent hindered the adsorption of atomic and ionic hydrogen at the reaction interface. On the other hand, after high-temperature plasma treatment, H_2_ produced high-energy excited-state active particles, such as H^+^, H_2_^+^, and H, which reduced the energy supply required.

**Figure 13:**
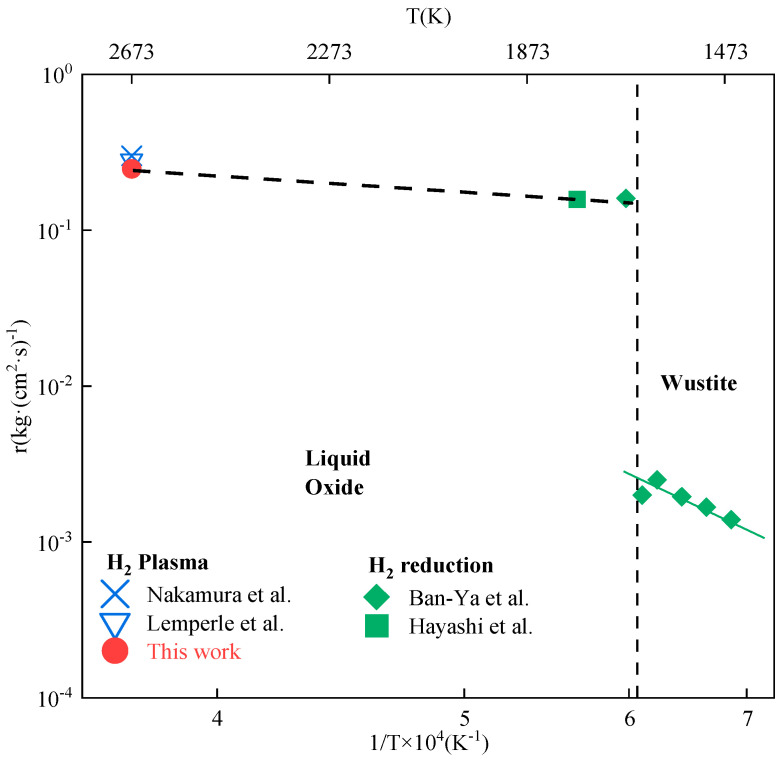
Variations in reduction rates of hydrogen-reduced ferrite and liquid iron oxide with temperature: Nakamura et al. [13], Lemperle et al. [30], Ban-Ya et al. [28], Hayashi et al. [31].

The activation energy is a significant indicator of the reaction difficulty that is directly connected to the temperature. During plasma injection into the crucible, there was a large temperature difference between the top and bottom. The plasma jet produced a substantial thermal shock effect on the top of the crucible, which provided adequate heat. The inside of the melt bath could be approximated as an isothermal region. Kaneko et al. [29] found that the average surface temperature of a plasma-heated iron melt bath was 2300 to 2350 K. Therefore, the plasma flame interacted with the melt bath at 2300 K as the real temperature. The reaction interface was formed by the contact surface between the plasma flame and the melt bath, with a 5 mm diameter arc-shaped flame region chosen as the reaction’s effective size. In this experiment, the specific reduction rate is defined using the conversion rate per unit area of the melt bath, this plasma molten reduction rate of acidic pellets was calculated to be 24.8 × 10^−2^ kg (m^2^ s)^−1^. This finding was comparable to the specific reduction rates found by Lemperle et al. [30] and Nakamura et al. [13] using identical experimental settings, which were 28 × 10^−2^ and 30 × 10^−2^ kg (m^2^ s)^−1^, respectively. Figure 13 shows the specific reduction rates of pure H_2_ and hydrogen plasma at various temperatures for molten FeO and wüstite based on the Ar-H_2_ mixed plasma molten reduction of iron oxides [13,30] and the average rate of H_2_ reduction of the molten iron oxides in this study. Fitting was performed to determine the relationship between the specific reduction rate and T^−1^.

In Figure 13, the points to the right of the dashed line are shown for H_2_ reduction which is termed “gas-based reduction”. The points to the left of the dashed line are shown for H_2_ plasma reduction and are termed “smelt reduction”. The slope of the fitted line corresponds to the value of −Ea/R, where Ea is the activation energy of the reaction, and R is 8.314 J (mol K)^−1^. For this experiment, the activation energy, calculated using the linear regression equation, was 21.9 kJ mol^−1^. The fitted equation is shown as follows:

(4) $$\ln r=-\frac{2637.95}{T}-0.407,$$

where r is the specific reduction rate (kg (m^2^ s)^−1^).

### 3.6. Phase Transformation Rules of Acidic Pellets Reduced by Hydrogen Plasma

**Figure 14:**
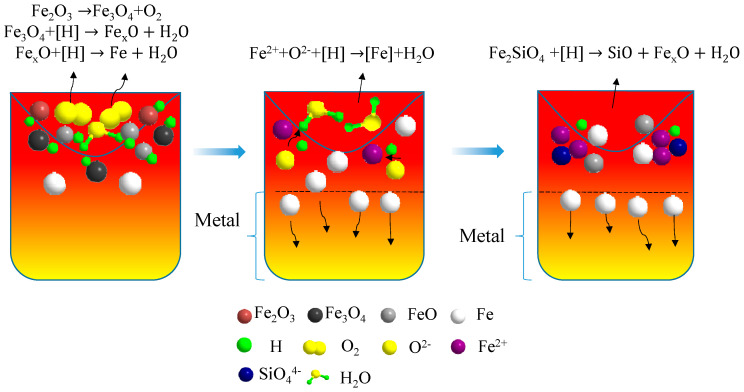
Diagram of metal phase transformation rules in hydrogen plasma reduction of acidic pellets.

The phase composition following the reaction was determined based on the conservation of elements before and after the reaction, as well as the XRD diffraction patterns shown in Figure 4. Based on the results, the hydrogen plasma reduction in this experiment was divided into the following stages. Figure 14 shows the diagram of the phase transformation characteristics in the hydrogen plasma reduction of acidic pellets.

(1) First stage: High-temperature melting of Fe_2_O_3_ and surface reduction by hydrogen plasma (t ≤ 10 min)

Acidic pellets were melted under high-temperature conditions with a plasma torch gas flow, generating a concave-downward melt bath. When heated, a small amount of Fe_2_O_3_ evaporated, and the remaining Fe_2_O_3_ on the surface of the melt bath reduced to Fe_3_O_4_. The gas flow caused the generated Fe_3_O_4_ to splash onto the inner walls of the crucible. Adsorption of atmospheric particles, such as H^+^ and H, on the surface of the Fe_3_O_4_ slag led to reduction processes. The reaction-produced Fe_x_O combined with slag system oxides such as SiO_2_, generating a trace amount of Fe_2_SiO_4_ in the spaces between unreduced Fe_x_O oxides. The main reactions occurring at this stage can be represented as follows:

(5) $${2Fe}_{2}O_{3}\to 4Fe\left(g\right)↑+3O_{2}↑,$$

(6) $${3Fe}_{2}O_{3}+2\left[H\right]\to {2Fe}_{3}O_{4}+H_{2}O\left(g\right)↑,$$

(7) $$\frac{x}{3}{Fe}_{3}O_{4}+\left(\frac{8x-6}{3}\right)\left[H\right]\to {Fe}_{x}O\left(l\right)+\left(\frac{4x-3}{3}\right)H_{2}O\left(g\right)↑,$$

(8) $${Fe}_{x}O+2\left[H\right]\to xFe(l)+H_{2}O(g)↑.$$

(2) Second stage: Rapid reduction of hydrogen plasma and effective deposition of metallic iron (10 min ≤ t ≤ 30 min)

Under the intense stirring action of high-temperature plasma gas flow, the core part of the melt bath had a concave-downward form. At this point, Fe_x_O and Fe_3_O_4_ in the crucible rapidly migrated to the reaction interface with the environment and adsorbed there. At high temperatures, Fe_x_O and Fe_3_O_4_ dissolved into free Fe^2+^ and O^2−^ ions. H^+^ and H particles in the environment permeated to the reaction interface and adsorbed on the reduction active sites of Fe_3_O_4_ and FexO. The melting point of Fe_3_O_4_ was lower than that of Fe_x_O (the melting point of Fe_x_O was 1377 °C). However, when the temperature surpassed the melting point of Fe_x_O, the number of reduction active sites on the Fe_x_O melt increased by roughly two orders of magnitude compared to that on Fe_3_O_4_. At this stage, the controlling step of the reaction was the diffusion process of H particles and iron oxides to the reaction interface. Furthermore, the ionic Si^4+^ tended to react with Fe_x_O, resulting in a small quantity of Fe_2_SiO_4_. Because of the density difference between the reaction-produced metallic iron and the molten slag, under the external force generated by the flame flow impact, the metallic iron tended to accumulate toward the outer ring area at the bottom of the crucible, effectively preventing it from entering the reaction core and triggering a reverse reduction reaction. The main reactions occurring at this stage can be represented as follows:

(9) $${Fe}^{2+}+O^{2-}+2\left[H\right]=\left[Fe\right]+H_{2}O(g)↑.$$

(3) Third stage: Slow reaction stage of Fe_x_O and Fe_2_SiO_4_ (t ≥ 30 min)

In the later stages of the reaction, some of the steam generated by the reduction reaction reacted at the phase boundary. In the acidic slag system used in this experiment, some steam decomposed to produce active hydrogen atoms [H] and oxygen ions O^2−^, which then permeated into the slag phase. These active particles rapidly reacted with the small amounts of Fe_x_O and Fe_2_SiO_4_, producing elemental Fe and products containing the Fe_x_O structure. During this process, an amount of Fe_2_SiO_4_ and Fe phases are physically blown out by high-speed jet impact. However, due to the high viscosity of Fe_2_SiO_4_, the fluidity of the slag system was significantly reduced, which hindered the mass transfer process of Fe_x_O to the reaction interface, causing the concentration of the reactant Fe_x_O to decrease. Therefore, the reaction controlling step in this stage mainly depended on the concentration of Fe_x_O in the slag phase and the mass transfer process of Fe_x_O and Fe_2_SiO_4_ to the reaction interface. The main reactions occurring at this stage can be represented as follows:

(10) $$\frac{x}{2}{Fe}_{2}SiO_{4}+(3x-2)\left[H\right]\to \frac{x}{2}SiO+{Fe}_{x}O+\left(\frac{3x-2}{2}\right)H_{2}O(g)↑$$

## 4. Conclusions

We used plasma heating of 15% hydrogen content under different Ar + H_2_ flow rates to achieve a non-transfer arc in the reduction of pellets at laboratory scale. This approach successfully yielded iron with a purity of 99.89%. The following conclusions were drawn.

(1) The hydrogen plasma reduction of acidic pellets generally exhibited a stepped trend. At a hydrogen concentration of 15%, the gas flow rate of 4.5 L min^−1^ achieved a conversion rate of 99.89% for acidic pellets in 40 min, while the gas flow rate of 3 L min^−1^ achieved a maximum of 82.5% reduction in 40 min. The deoxygenation rate per area increased from 0.87 g (cm^2^ min)^−1^ at 3 L min^−1^ to 1.11 g (cm^2^ min)^−1^ at 4.5 L min^−1^. This suggested that it is possible to achieve a high reduction rate of acidic pellets with lower hydrogen concentrations and higher gas flow rates.
(2) The hydrogen plasma reduction of acidic pellets followed a three-dimensional diffusion-controlled reaction model, with the controlling step primarily being the diffusion of the reactants Fe^2+^, [H], and O^2−^ particles toward the reaction interface. The mechanism function was determined to be 2/3(1 + α)^3/2^[(1 + α)^1/3^ − 1]^−1^. Under different gas flow rates (3 and 4.5 L min^−1^), the corresponding reduction reaction rate constants k(T) were 12.6 × 10^−2^ and 12.8 × 10^−2^ s^−1^, respectively. The apparent activation energy was 21.9 kJ mol^−1^, and the empirical equation for the reaction reduction rate was ln r = −2637.5/T − 0.407.
(3) After the acidic pellets underwent HPSR, the contents of impurity elements such as Si and Al could be effectively reduced. After 40 min of reduction, the metallic iron achieved a purity of 99.90%, meeting the purity requirements for industrial 3N-grade high-purity iron. The findings of this study can accelerate the application of hydrogen plasma in the metallurgical industry and provide a theoretical basis for developing new technologies in the steel industry.

## Figures and Tables

**Table 1:** Chemical composition of acidic pellets (%).

Fe_tot_	Si	Al	Ti	Mg	V	Mn	Ca	Ni	P	S	Zn	O
69.35	0.84	0.41	0.34	0.27	0.13	0.12	0.12	0.02	0.02	0.02	0.01	28.35

**Table 2:** Experimental parameters of acidic pellets for hydrogen plasma reduction.

Group	Gas Composition	Ar Flow Rate (L min^−1^)	H_2_ Flow Rate (L min^−1^)	Reduction Time (min)
1	Ar + 15%H_2_	17	3	10, 20, 30, 40
2	Ar + 15%H_2_	25.5	4.5	10, 20, 30, 40

**Table 3:** Elemental analysis results of points 1 to 4 in Figure 5 (%).

Point	wt/%					
	Fe	O	Si	Al	Mg	Ca
Point 1	51.16	34.50	6.15	4.13	1.00	3.06
Point 2	81.18	17.97	0.12	0.61	0.10	0.01
Point 3	99.62	0.32	0.04	0.01	0.00	0.01
Point 4	99.85	0.12	0.03	0.00	0.00	0.00

**Table 4:** Elemental analysis results at points 1 to 3 in Figure 8 (%).

Point	wt/%					
	Fe	O	Si	Al	Mg	Ca
Point 1	54.79	22.16	12.65	8.37	1.09	0.84
Point 2	85.54	13.71	0.36	0.40	0.16	0.03
Point 3	99.87	0.36	0.07	0.00	0.00	0.00

**Table 5:** Maximum reduction rate, maximum reduction rate, and deoxygenation rate per area for different reduction atmospheres and reduction times.

Gas Flow Rate (L min^−1^)	Maximum Conversion Rate (%)	Deoxygenation Rate per Area (g (cm^2^ min)^−1^)
17Ar + 3H_2_	82.67%	0.87
25.5Ar + 4.5H_2_	99.89%	1.11

## Data Availability

The original contributions presented in this study are included in the article. Further inquiries can be directed to the corresponding authors.
